# 

**Published:** 1863-01

**Authors:** 


### MEDICAL CRITIC

AND

### PSYCHOLOGICAL

### JOURNAL.

EDITED BY

FORBES WINSLOW, M.D., D.C.L. Oxon.

VOL. III.

The last.

(See preface)

60772

LONDON:
JOHN W. DAVIES, 4, PRINCES STREET,
LEICESTER SQUARE.
EDINBURGH: MACLACHLAN AND CO. DUBLIN: FANNIN AND CO.

MDCCCLXIII.

WI
ME 286

LONDON:
SAVILL AND EDWARDS, PRINTERS, CHANDOS STREET,
COVENT GARDEN.

WITH this number of the "JOURNAL OF PSYCHOLOGICAL MEDICINE" I resign the editorial *baton*. The questions as to its amalgamation with another magazine, or its subsequent re-appearance in a somewhat different form, are reserved for future consideration.

For sixteen years I have edited this Journal. It was established by myself in January, 1848. As it was the first periodical work exclusively devoted to the discussion of medico-psychological literature published in this country, my readers will easily appreciate the many difficulties with which I had to contend at the early period of its existence, and the grave and responsible duties that devolved upon me, during at least the first few years of my editorial career.

Until within a recent period the sole management of this Journal was in my own hands. For ten years I discharged the onerous functions of editor, sub-editor, writing many of the original articles and reviews, revising the original articles forwarded for insertion, correcting the proof-sheets, and personally superintending all the complex mechanical arrangements of the press. If this had not been to me a labour of love I never should have been able, with my other numerous and anxious engagements, to have undergone for so many years this serious amount of mental work with impunity.

In 1858 I found it necessary, in consequence of my increasing professional engagements, to appoint a sub-editor. For this responsible position I was fortunate in being able to secure the literary services of Mr. J. N. RADCLIFFE, who, as all my readers as well as myself can testify, has discharged his duties with great ability.

Before bidding a final editorial adieu, I desire to proffer my warm and sincere thanks to the many learned contributors who have, from the commencement of this Journal, rendered me most invaluable literary assistance; and of the generous and liberal support I have, almost without an exception, received from the public and medical press of this country, I cannot speak in terms sufficiently expressive of my gratitude.

If in the course of my editorial reign anything has been inadvertently said in this Journal to give pain or annoyance to any person, I now desire, in the most unqualified terms, to express my regret.

I have, during the whole period of my connexion with this periodical, endeavoured to act kindly and conscientiously towards all parties—studiously, as far as was consistent with just criticisms, avoiding all personalities.

Some of my readers may, however, conceive that criticisms which these pages have set forth have occasionally been harsh or even unjust. I do not wish it to be inferred that observations have not occurred in some of the articles which it would have been better to have left unsaid.

I have no desire to refer to this Journal as a standard of critical perfection, or to claim for myself, as its editor, an exemption from the ordinary frailties that are, alas! so closely interwoven with poor human nature.

Errors have no doubt been committed that I regret, and remarks made which I would willingly (if it were in my power) expunge; but—

"To err is human, to forgive divine."

It would be egotistical for me to speak of the increased interest that has been awakened in all medico-psychological questions (evidenced by the establishment of a Professorship of Psychological Medicine, and the publication within the last ten years of numerous works on the subject of psychology in relation to the science and practice of medicine); or of the enlightened progress made in the treatment of the insane, arising out of a more scientific appreciation of the great principles of mental and cerebral pathology.

The relation between these matters and the publication for sixteen years of this work must be left to the judgment of unbiassed minds and the testimony of disinterested persons.

In conclusion, I wish to intimate to my numerous readers and subscribers that immediate steps will be taken to bind up the first and second series of this journal, now embracing sixteen closely-printed volumes.

It is also my intention to prepare for speedy publication a copious index of both series, in one octavo volume. This will be found invaluable to all who already possess copies of the work, and will also be highly useful to those, unconnected with the medical profession, engaged in the study of this deeply-interesting and important branch of philosophical inquiry.*

FORBES WINSLOW.

BELLAGIO, LAKE OF COMO,

*Sept.* 19*th*, 1863.

---

* As a limited number of this index-volume will be printed, all who wish to enrol their names as subscribers may do so at the publishers.

*Pelvic Hæmatocele*, by J. Byrne, M.D., Resident Fellow of the New York Academy of Medicine, &c. (New York.) Mr. Yearsley's *Introduction to the Art of Laryngoscopy*, reprinted from the *Medical Circular;* an Essay *On the Causes of the Evils incident to Infant Dentition*, by J. C. Clendon, M.R.C.S.; and *An Appeal to Physiologists and the Press*, by H. Freke, A.B., M.B.

### WORKS PUBLISHED DURING THE QUARTER.

### 1. *Medicine.*

BEGBIE.—Contributions to Practical Medicine. By James Begbie, M.D. 8vo, cloth, 10s. 6d.

BENNET.—Mentone, the Riviera, Corsica, and Biarritz, as Winter Climates. By J. Henry Bennet, M.D. Second Edition, much Enlarged. Post 8vo, cloth, 5s.

BOWER HARRISON.—Letters to a Young Practitioner on the Diseases of Children. By J. Bower Harrison, M.D., M.R.C.P. Fcap. 8vo, cloth, 3s.

BRODIE.—Psychological Enquiries. Part I. Fourth Edition. Fcap. 8vo, cloth, 5s.

CARPENTER.—The Microscope and its Revelations. By William B. Carpenter, M.D., F.R.S. Third Edition, Plates, Fcap. 8vo, cloth, 12s. 6d.

CHAMBERS.—The Renewal of Life: Clinical Lectures Illustrative of a Restorative System of Medicine. By T. K. Chambers, M.D. Post 8vo, cloth, 6s. 6d.

FLEISCHMANN.—Plain and Practical Medical Precepts. By Alfred Fleischmann, M.R.C.S. Second Edition, Revised and much Enlarged. On a Large Sheet, 4d.

FULLER.—On Diseases of the Chest, including Diseases of the Heart and Great Vessels. By Henry Wm. Fuller, M.D. Cantab., F.R.C.P. 8vo, cloth, 12s. 6d.

GORDON.—China, from a Medical Point of View in 1860 and 1861, to which is added a Chapter on Nagasaki as a Sanitarium. By Charles Alexander Gordon, M.D., and C.B. With Plans, 8vo, cloth, 10s. 6d.

GUY'S HOSPITAL.—Guy's Hospital Reports. Edited by S. Wilks, M.D., and A. Poland, F.R.C.S. New Series. Vol. VIII. 8vo, 7s. 6d.

LEARED.—Infant Mortality and its Causes. By Arther Leared, M.D., M.R.I.A. 8vo, sewed, 6d.

MACPOTHER.—Physiology, and its Aids to the Study and Treatment of Disease. With Illustrations, Examination Papers, and a Glossary of Medical Terms. By E. Dillon Macpother. Fcap. 8vo, 9s.

MARCET.—On Chronic Alcoholic Intoxication; with an Inquiry into the Influence of the Abuse of Alcohol as a Predisposing Cause of Disease. By W. Marcet, M.D., F.R.S., F.R.C.P. Second Edition, Enlarged, Fcap. 8vo, cloth, 4s. 6d.

MEDICO-CHIRURGICAL SOCIETY.—Transactions Published by the Royal Medical and Chirurgical Society of London. Vol. XLV. Second Series, Vol. XXVII. 8vo, cloth, 16s.

MAYNE.—A Medical Vocabulary; or, an Explanation of all Names, Synonymes, Terms, and Phrases used in Medicine and the Relative Branches of Medical Science; intended specially as a Book of Reference for the Young Student. By R. G. Mayne, M.D. Fcap. 8vo, cloth, 8s. 6d.

MOORE.—Health in the Tropics; or, Sanitary Art Applied to Europeans in India. By W. J. Moore, L.R.C.P. Edin. 8vo, cloth, 9s.

MURCHISON.—A Treatise on the Continued Fevers of Great Britain. By Charles Murchison, M.D., F.R.C.P., &c. Coloured Plates and Diagrams. 8vo, cloth, 18s.

OBSTETRICUS.—On the Evils resulting from Rising too early after Childbirth. By Obstetricus. 8vo, sewed, 6d.

PATHOLOGICAL SOCIETY.—The Transactions of the Pathological Society of London. Vol. XIII. Comprising the Report of the Proceedings for the Session 1861-62. 8vo, cloth, 12s. 6d.

PRIESTLEY.—Rational Medicine, its Objects and Study. Introductory Address at the Middlesex Hospital Medical College, 1862. By William O. Priestley, M.D. 8vo, sewed, 6d.

SAVORY.—A Compendium of Domestic Medicine, and Companion to the Medicine Chest. By John Savory, M.S.A. Sixth Edition, post 8vo, cloth, 5s.

SHAPTER.—The Climate of the South of Devon, and its Influence upon Health. By Thomas Shapter, M.D., F.R.C.P. Second Edition, Maps, 8vo, cloth, 10s. 6d.

TOWNLEY.—Parturition without Pain or Loss of Consciousness. By James Townley, L.R.C.P. Edin., F.L.S. Second Edition, post 8vo, cloth, 2s. 6d.

TUERCK.—Clinical Researches on Different Diseases of the Larynx, Trachea, and Pharynx. Examined by the Laryngoscope, &c. By Lewis Tuerck, M.D. 8vo, sewed, 3s.

TWEEDIE.—Continued Fevers: their Distinctive Characters, Pathology, and Treatment. By Alexander Tweedie, M.D., F.R.S. Coloured Plates. 8vo, cloth, 12s.

WATERS.—Researches on the Nature, Pathology, and Treatment of Emphysema of the Lungs, and its Relations with other Diseases of the Chest. By A. T. H. Waters, M.D., M.R.C.P. Plates, 8vo, cloth, 5s.

UVEDALE WEST.—Illustrations of Puerperal Diseases. By R. Uvedale West, M.D. Second Edition, Enlarged, post 8vo, cloth, 5s.

### 2. *Surgery.*

ST. BARTHOLOMEW'S HOSPITAL.—A Descriptive Catalogue of the Anatomical Museum of St. Bartholomew's Hospital. Vol. III. 8vo, cloth, 5s.

LAWRENCE.—Lectures on Surgery. By William Lawrence, F.R.S. 8vo, cloth, 16s.

ERASMUS WILSON.—Diseases of the Skin; a Practical and Theoretical Treatise on the Diagnosis, Pathology, and Treatment of Cutaneous Diseases. By Erasmus Wilson, F.R.S. Fifth Edition, revised, 8vo, cloth, 16s.; with Plates, 34s.

YEARSLEY.—Deafness Practically Illustrated, being an Exposition as to the Nature, Causes, and Treatment of Diseases of the Ear. By James Yearsley, M.D., M.R.C.S. Sixth Edition, with Engravings. 8vo, cloth, 6s.

### 3. *Chemistry.*

BOWMAN.—Bowman's Medical Chemistry. Edited by Charles L. Bloxam. Engravings. Fourth Edition, fcap. 8vo, cloth, 6s. 6d.

BRANDE AND TAYLOR.—Chemistry. By William Thomas Brande, D.C.L., F.R.S.L. & E., &c., and Alfred Swaine Taylor, M.D., F.R.C.P. Lond., F.R.S., &c. Fcap. 8vo, cloth, 12s. 6d.

CONDY.—Air and Water, their Impurities and Purification. By Henry Bollman Condy. 8vo, cloth, 3s. 6d.

